# Leucinostatins from fungal extracts block malaria transmission to mosquitoes

**DOI:** 10.1186/s13071-024-06450-y

**Published:** 2024-09-20

**Authors:** Guodong Niu, Xiaohong Wang, Wenda Gao, Liwang Cui, Jun Li

**Affiliations:** 1 Department of Biological Sciences, Florida International University, Miami, FL 33199 USA; 2 Antagen Pharmaceuticals, Canton, MA 02021 USA; 3 Department of Internal Medicine, Morsani College of Medicine, University of South Florida, Tampa, FL 33612 USA

**Keywords:** Malaria, Mosquito, Leucinostatin, Infection, Transmission, Transmission-blocking, Multiple-stage antimalarials, External spray

## Abstract

**Background:**

Malaria is a mosquito-transmitted disease that kills more than half a million people annually. The lack of effective malaria vaccines and recently increasing malaria cases urge innovative approaches to prevent malaria. Previously, we reported that the extract from the soil-dwelling fungus *Purpureocillium lilacinum*, a common fungus from the soil, reduced *Plasmodium falciparum* oocysts in *Anopheles gambiae* midguts after mosquitoes contacted the treated surface before feeding.

**Methods:**

We used liquid chromatography to fraction fungal crude extract and tract the active fraction using a contact-wise approach and standard membrane feeding assays. The purified small molecules were analyzed using precise mass spectrometry and tandem mass spectrometry.

**Results:**

We isolated four active small molecules from *P. lilacinum* and determined them as leucinostatin A, B, A2, and B2. Pre-exposure of mosquitoes via contact with very low-concentration leucinostatin A significantly reduced the number of oocysts. The half-maximal response or inhibition concentration (EC_50_) via pre-exposure was 0.7 mg/m^2^, similar to atovaquone but lower than other known antimalarials. The inhibitory effect of leucinostatin A against *P. falciparum* during intraerythrocytic development, gametogenesis, sporogonic development, and ookinete formation, with the exception of oocyst development, suggests that leucinostatins play a part during parasite invasion of new cells.

**Conclusions:**

Leucinostatins, secondary metabolites from *P. lilacinum* disrupt malaria development, particular transmission to mosquitoes by contact. The contact-wise malaria control as a nonconventional approach is highly needed in malaria-endemic areas.

**Graphical Abstract:**

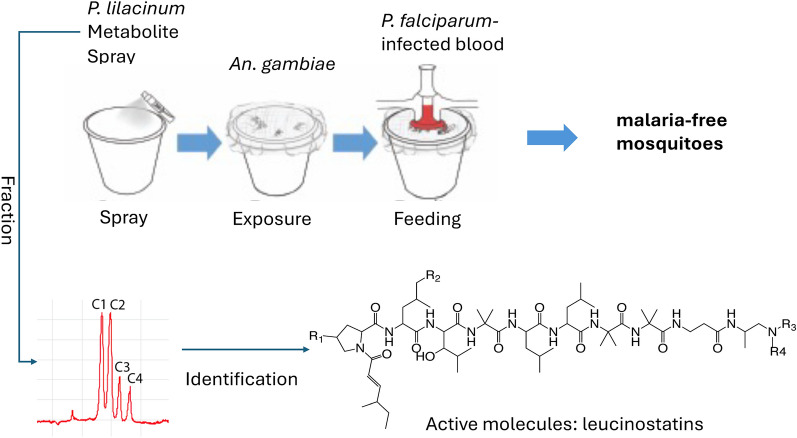

**Supplementary Information:**

The online version contains supplementary material available at 10.1186/s13071-024-06450-y.

## Background

Malaria, a major tropical disease transmitted by *Anopheles* mosquitoes, remains a critical health problem. There were more than 247 million malaria cases and about 619 000 malaria deaths worldwide in 2022 [1]. Combating against malaria faces many challenges, including the emergence and spread of *Plasmodium* spp. parasites resistant to currently used antimalarial drugs [2, 3], modest protective vaccines [4], and the widespread and increasing insecticide resistance in major malaria vectors [5]. Novel strategies are needed to achieve worldwide malaria control and elimination.

Because transmission through mosquitoes is the obligatory step during the malaria life cycle, reducing mosquitoes population density or inhibiting parasite infection in mosquitoes will break malaria transmission. Biocontrol of mosquito populations through entomopathogenic fungi is one of the recent research areas for vector control [6, 7]. We focus on natural products that can prevent parasites from infecting mosquitoes. Previous studies show that malaria transmission entails extensive interactions between parasites and mosquitoes [8–12]. Such interactions during midgut invasion have been explored as vaccine targets for blocking malaria transmission [9, 13–15]. Drug-based approaches for blocking parasite transmission have also received tremendous attention [16–18].

In 2016, we proposed a concept that pre-exposing mosquitoes with fungal secondary metabolites via environmental contact could prevent mosquitoes from *Plasmodium* infection. Encouragingly, our initial screening from our fungal library [19] for transmission-blocking (TB) effects demonstrated that pre-exposing adult female *An. gambiae* with a trace amount of *P. lilacinum* extract had significantly fewer *P. falciparum* oocysts [20], providing the basis for using environmental applications of fungal secondary metabolites as a novel strategy for interrupting malaria transmission.

This study further examined this new approach of using external nontoxic chemicals to inhibit the development of malaria pathogens in mosquitoes. Furthermore, we isolated and identified a set of active natural products from *P. lilacinum*; pre-exposing mosquitoes to these natural compounds prevented parasites from developing into oocysts in midguts.

## Methods

### Maintenance of *An. gambiae*

The mosquitoes (*An. gambiae* G3 strain) were maintained with cotton balls soaked with 8% sugar in an insectary at 27 °C, 80% humidity, and a 12-h day/night cycle. The 3–10-day-old female adults were fed with human blood (Continental Blood Bank, FL) using glass feeders (chemglass, NJ) for egg production. The blood engorged mosquitoes were maintained with 8% sugar. On day 2 post-bloodmeal, a filter paper soaked in a de-ionized-water beaker was placed into the cage to collect eggs. Next day, the filter paper was placed into DI-water. The hatched larvae were fed with 0.05 mg/larva of ground KOI fish food.

### Culturing *P. falciparum*

*P. falciparum* (NF54) was obtained from the American Type Culture Collection (ATCC) and maintained as described previously [14]. In brief, the frozen infected blood from liquid nitrogen was thawed at room temperature (RT) for 5 min and collected by centrifugation (300 × g for 3 min). About 50–100 μl cells were inbuated with 3% NaCl at RT. After removing 3% NaCl by centrifugation, the cells were washed with RMPI-1640 and collected by centrifugation (300 × g for 3 min). After being washed three times, cells were maintained in a 5 mL complete RPMI-1640 (named after Roswell Park Memorial Institute) medium containing 4% fresh O^+^ human red blood cells, 10% human heat-inactivated AB^+^ serum (56 °C for 30 min), and 12.5 μg/mL of hypoxanthine in a candle jar at 37 °C as described previously [16, 20]. The complete RPMI-1640 medium was replaced daily. To prepare *P. falciparum* gametocytes for infecting mosquitoes, the parasites were maintained until the culture containing > 2% stage-V gametocytes, which took about 15 days.

### Preparation of fungal extracts

**Fig. 1:**
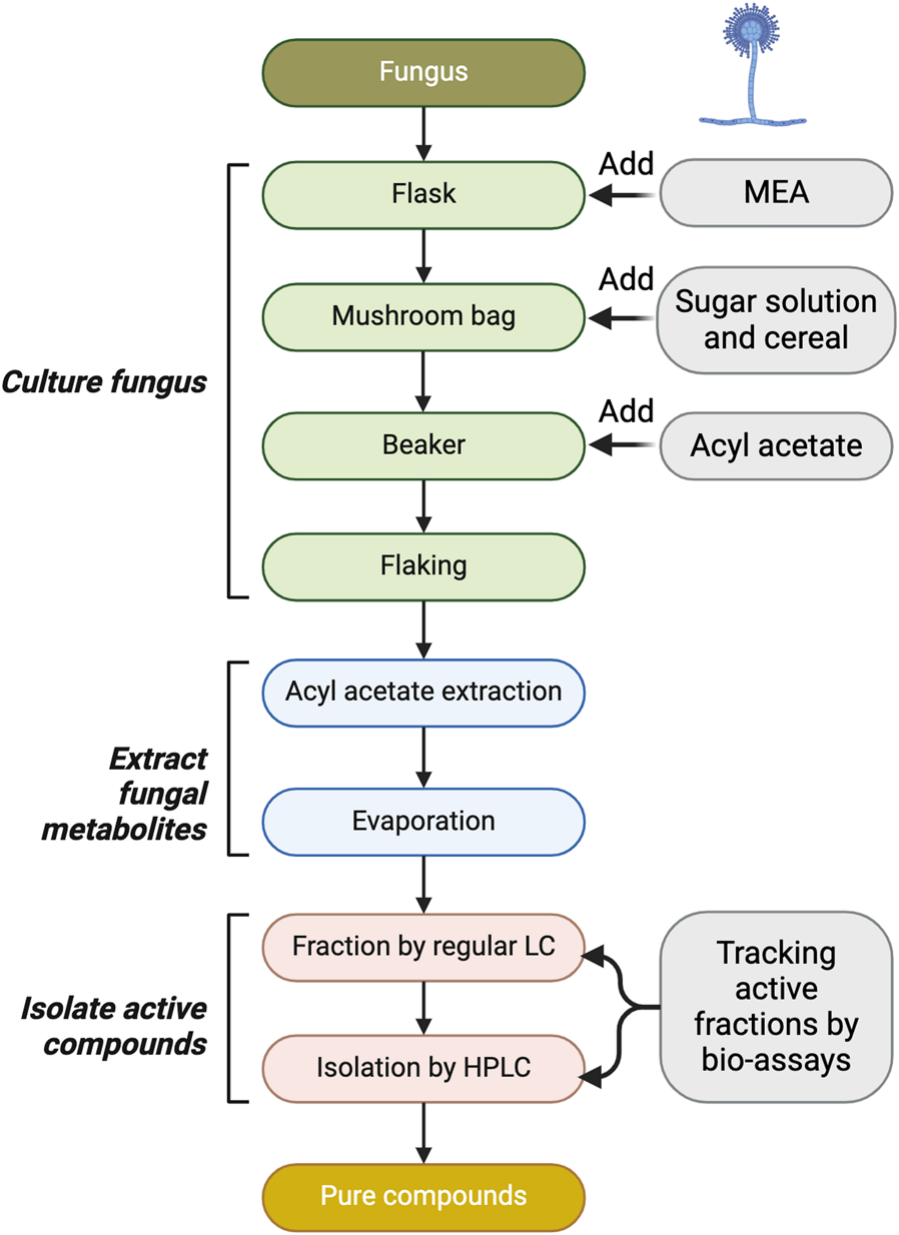
Preparation protocol of fungal extracts

Fungal extracts were prepared as previously described [20]. The protocol is shown in Fig. 1. Briefly, a *P. lilacinum* seed culture was prepared by inoculating the fungus in a malt extract liquid medium (MEA) consisting of 5 g of malt extract, 7.5 g of agar, and 0.025 g of chloramphenicol in 1 L of distilled water, which was shaken for 4 days at 25 °C. A sugar solution was prepared with 3 g of sucrose and 50 mg of chloramphenicol in 1 L of distilled water in conical flasks and autoclaved for 15 min. An autoclavable plastic bag with a 0.22 μm filter (mushroom bag) was filled with 550 g of autoclaved Cheerios cereal (General Mills Cheerios Cereal) as the carbon source for fungal fermentation. The sugar solution, cereal, and the 50 mL seed fungal culture were mixed in the mushroom bag, and the culture was fermented for 4 weeks at 25 °C with a 12-h day/night cycle. Afterward, the fermented product was transferred to a glass container, and two volumes of ethyl acetate were added for extraction for 24 h. The supernatant was collected and filtered through a Buchner funnel lined with filter paper. Two additional 500 mL of ethyl acetate were added for extraction for 4 h. The ethyl acetate extract was dried using a rotary vacuum evaporator at room temperature.

### Isolation of the active fungal metabolites

About 20 g of *P. lilacinum* extract were dissolved in 100 ml methanol and mixed with 40 g silica gel (230–400 mesh, 40–63 µm) (Sorbent Technologies, Norcross, GA, USA). After drying, the sample was applied to a silica gravity column using a sintered glass funnel (90 × 250 cm) packed with 200 g of silica gel (230–400 mesh, 40–63 µm). A total of six fractions were collected, each at four volumes of the silica gel (~ 1.6 L), with the following solvents: 100% hexane (F1), 100% dichloromethane (DCM, F2), 90:10 (DCM:Methanol, F3), 80:20 (DCM:Methanol, F4), 50:50 (DCM:Methanol, F5), and 100% Methanol (F6). The collected fractions were dried over a vacuum and tested for malaria TB activity using the spray-exposure method. The active F4 fraction was analyzed with reverse-phase high-performance liquid chromatography (HPLC) (Shimadzu LC-20AD pump, SPD-M20A detector, and FRC-10A fraction collector, Shimadzu, Columbia, MD, USA). A semi-preparative column (Gemini C18 250 × 10 mm, 5 µm, Phenomenex, Torrance, CA, USA) was used with a flow rate of 5 mL/min by a linear gradient solvent of acetonitrile and H_2_O containing 0.2% trifluoroacetic acid from 10% acetonitrile to 100% acetonitrile. The flow rate was 5 mL/min, and the total time was 35 min. The eluted fractions based on the retention time were collected, and their activities were determined using the spray-exposure method.

### Liquid chromatography-mass spectrometry (LC–MS) and tandem mass spectrometry (MS/MS) analysis

All the active fractions were purified using the ultra-high-performance (UHP) Accurate-Mass Q-TOF LC–MS with a 1260 HPLC System (Agilent Technologies, Santa Clara, CA). It consisted of a UHP chromatography system (UPLC-Waters) equipped with a HiP autosampler, G1312B binary pump, and a Luna C18(2) (100 × 4.6 mm, 5 mm, Phenomenex, Torrance, CA, USA). The mass spectrometer was run with Bruker Q-TOF analysis and operated with ESI in the positive mode, in the scan range m/z 110–1700. The solvents employed in the elution were mobile phase A (acetonitrile:H_2_O containing 0.1% formic acid; 2:98) and mobile phase B (acetonitrile:H_2_O containing 0.1% formic acid; 98:2). The chromatography started with 100% phase A and increased linearly to 100% phase B within 15 min with a gradient mode with a flow rate was 0.5 ml/min.

The purified fungal secondary metabolites were further analyzed using MS/MS analysis using Bruker Solarix 7.0 T. Fragmentation data were processed utilizing the Bruker data analysis software package for the calculation of the m/z values of the collision induced dissociation (CID) fragments (b and y ions) and electron capture dissociation (ECD) fragments (c and z ions). Perkin Elmer’s ChemDraw suite was used to draw the chemical structures.

### Bioassays to determine contact-wise malaria transmission-blocking (TB) activity

Quinine, primaquine, artemisinin, artemether, arteether, artesunate, pyrimethamine, sulfadoxine, methylene blue, salinomycin (an antimicrobial and anticoccidial antibiotic), and atovaquone were purchased from Sigma (Millipore-Sigma, St. Louis, MO). Asperaculane B was previously isolated from the *Aspergillus aculeatus* [18].

The *P. lilacinum* extract, leucinostatins, and other antimalarial compounds were dissolved in DMSO. About 10 μL solution was diluted into 1 ml acetone in a 2-mL spray bottle. Drugs in acetone were sprayed onto the interior wall of a 16 oz paper cup (Solo Cup Company). The cups were dried at room temperature (RT) for about 1 h. The same amount of solvent DMSO diluted in acetone was used as the control. Then, about 80 mosquitoes were transferred into the cup and exposed to the sprayed internal wall for 1 h, and the mosquitoes were fed with human blood containing approximately 0.2% stage-V *P. falciparum* gametocytes for 30 min using standard membrane feeders. Engorged mosquitoes were maintained on 8% sugar in untreated cups in the insectary (27 °C, 80% humidity, 12-h day/night cycle). Mosquitoes were dissected 7 days after feeding, and midguts were stained with 0.1% mercury dibromofluorescein disodium salt in phosphate-buffered saline (PBS). Oocysts were counted under a light microscope. The number of oocysts in individuals and the average number of oocysts were summarized using prism software (GraphPad version 8.4.3).

### In vitro inhibitory activities of fungal metabolites against blood stage parasites

We used schizont maturation assay to determine the activity of a sample against the asexual stage of parasites [21]. In brief, we mixed cultured *P. falciparum*-infected red blood cells (iRBCs) with fresh human RBCs (AB^+^ type) in a complete medium to prepare cultures with 0.5% parasitemia and 2% hematocrit. A compound was dissolved in DMSO at a concentration of a series of dilutions from 0.1 ng/mL to 1 µg/mL. A 1 µL diluted compound was mixed with 0.2 mL parasite culture and seeded in a 96-well plate. The plate was incubated in a candle jar at 37 °C. Approximately 48 h later, the medium was replaced with a fresh medium containing the same concentration of the compounds. Parasitemia was determined at 72 h post-incubation by blood smear.

### In vitro inhibitory activities of fungal metabolites against gametocyte viability

Parasite infection of mosquitoes needs viable gametocytes. We used gametocyte viability assays to examine the activity of a compound against gametocyte development. The iRBCs containing 0.5% parasitemia were incubated at 37 °C in an atmosphere of 5% CO_2_ for 4 days. By then, the gametocytes were induced, and leucinostatin A (LA) at 150 ng/mL and 1.5 µg/mL was added to the culture. The medium was replaced daily with fresh medium containing the same concentration of the compound. Gametocytemia, including all stages of gametocytes, was recorded on day 14.

### Inhibitory activities of fungal metabolites against parasites in midguts by feeding

We used the standard membrane feeding assays (SMFA) [22] to determine the activity of a compound in preventing parasites from infecting mosquitoes. This process includes the egress of gametocytes, maturation of gametes, fertilization of microgametes and macrogametes, the transformation of zygotes to ookinetes, and ookinete invasion of mosquito midguts. Briefly, the 15- to 17-day-old cultured *P. falciparum* iRBCs containing 2–3% stage-V gametocytes were collected and diluted with fresh O^+^ type human blood and the same volume of heat-inactivated AB + human serum to obtain a final parasitemia of approximately 0.2%. Then, 1.5 μL of the tested compounds with varying concentrations in DMSO was mixed with 298.5 μL of gametocyte culture, which was used to feed sixty 3- to 5-day-old *An. gambiae* female mosquitoes for 30 min. Engorged mosquitoes were maintained with 8% sugar in a BSL-2 insectary. Oocysts were counted as described above.

We also examined leucinostatin A on the developed oocysts in mosquito midguts. About 100 3- to 5-day-old female mosquitoes were infected with *P. falciparum* by membrane feeding. We maintained mosquitoes with 10% sucrose. From day 2 to day 7, we fed mosquitoes with 10% sucrose containing 100 nM leucinostatin A daily and examined the shape and the number of oocysts in mosquito midguts under a microscope.

### Inhibitory activities of fungal metabolites against Plasmodium sporozoites in mosquito salivary glands

Only the living sporozoites in mosquito salivary glands infect humans when mosquitoes bite them. Thus, we examined the effect of leucinostatin A on sporozoites. We infected 100 3–5-day-old female mosquitoes with *P. falciparum*-infected blood through membrane feeding and maintained the engorged mosquitoes on 10% sucrose for 10 days. From day 11, we fed mosquitoes with 10% sugar containing 1 µg/mL leucinostatin A for 2 days. On day 13, we isolated sporozoites from mosquito salivary glands as described previously [23]. The viable sporozoites were counted under a light microscope.

### Statistical analysis

The nonparametric statistical analysis of the Wilcoxon–Mann–Whitney test was used to calculate the *P*-value for the difference in infection between control and experimental groups in each assay with Prism (GraphPad Software, CA, USA). The EC_50_ was determined by analyzing the dose–response curve online (AAT Bioquest, https://www.aatbio.com/tools/lc50-calculator).

## Results

### Contact-wise TB activity of *P. lilacinum* extract in mosquitoes

**Fig. 2:**
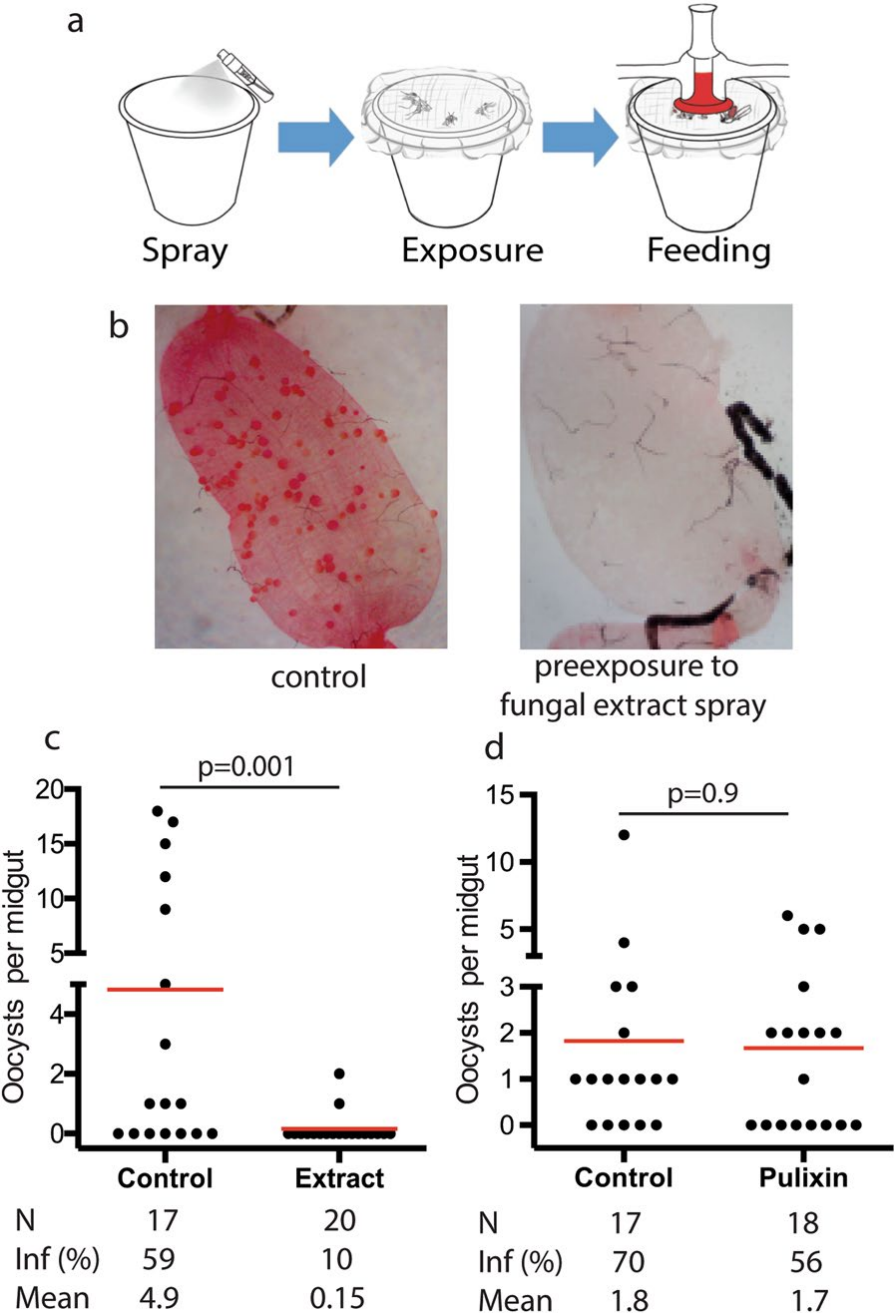
Pre-exposure of *P. lilacinum* extract blocked *P. falciparum* transmission to mosquitoes. **a** Outline of spraying approach. **b **The midguts of *P. lilacinum* extract-treated mosquitoes (right) had fewer oocysts than those of the control (left). Red dots inside the midguts are oocysts. **c** Pre-exposure of the fungal extract to *An. gambiae* at 40 mg/m^2^ significantly reduced the number of the oocyst in the midgut compared with the control (*P* = 0.0001). **d** Pre-exposure of pulixin, a previously identified secondary metabolite from *P. lilacinum*, to *An. gambiae* at 10 mg/m^2^ did not reduce *P. falciparum* oocysts in mosquitoes (*P* = 0.9), supporting that pulixin is not the active compound responsible for malaria transmission inhibition via contact. In c and d, N indicates mosquitoes dissected, Inf (%) indicates % infected mosquitoes and Mean shows the mean oocyst density (oocysts/midgut)

The extract from the *P. lilacinum* GFEL-12E6 isolate showed potent TB activity by feeding at 1 µg/mL [20]. We explored additional delivery methods using the spray-exposure as showed in Fig. 2a. Results showed that mosquitoes that pre-exposed with *P. lilacinum* extract had much fewer oocysts than the control (Fig. 2b). Exposure to 40 mg/m^2^ of the *P. lilacinum* extract rendered mosquitoes nearly free of oocysts (10% prevalence) and lower oocyst density (0.15 oocyst.midgut), compared with about 60% of oocyst prevalence and higher oocyst density (4.9 oocysts/midgut) in the control group (*P* = 0.0001 by Wilcoxon–Mann–Whitney test) (Fig. 2c).

Previously, we reported that pulixin from the *P. lilacinum* extract inhibited *P. falciparum* transmission when fed to mosquitoes [20]. The content of pulixin in the *P. lilacinum* extract is less than 5%. We mesured the contact-wise TB activity of pulixin at 10 mg/m^2^, equivalent to more than 200 mg/m^2^ of *P. lilacinum* extract. The results showed that 10 mg/m^2^ of pulixin did not reduce the number of oocysts by contact (*P* = 0.9, Fig. 2d), indicating that other *P. lilacinum* secondary metabolites are responsible for the contact-wise TB activity.

### Isolation of TB-active metabolites

**Fig. 3:**
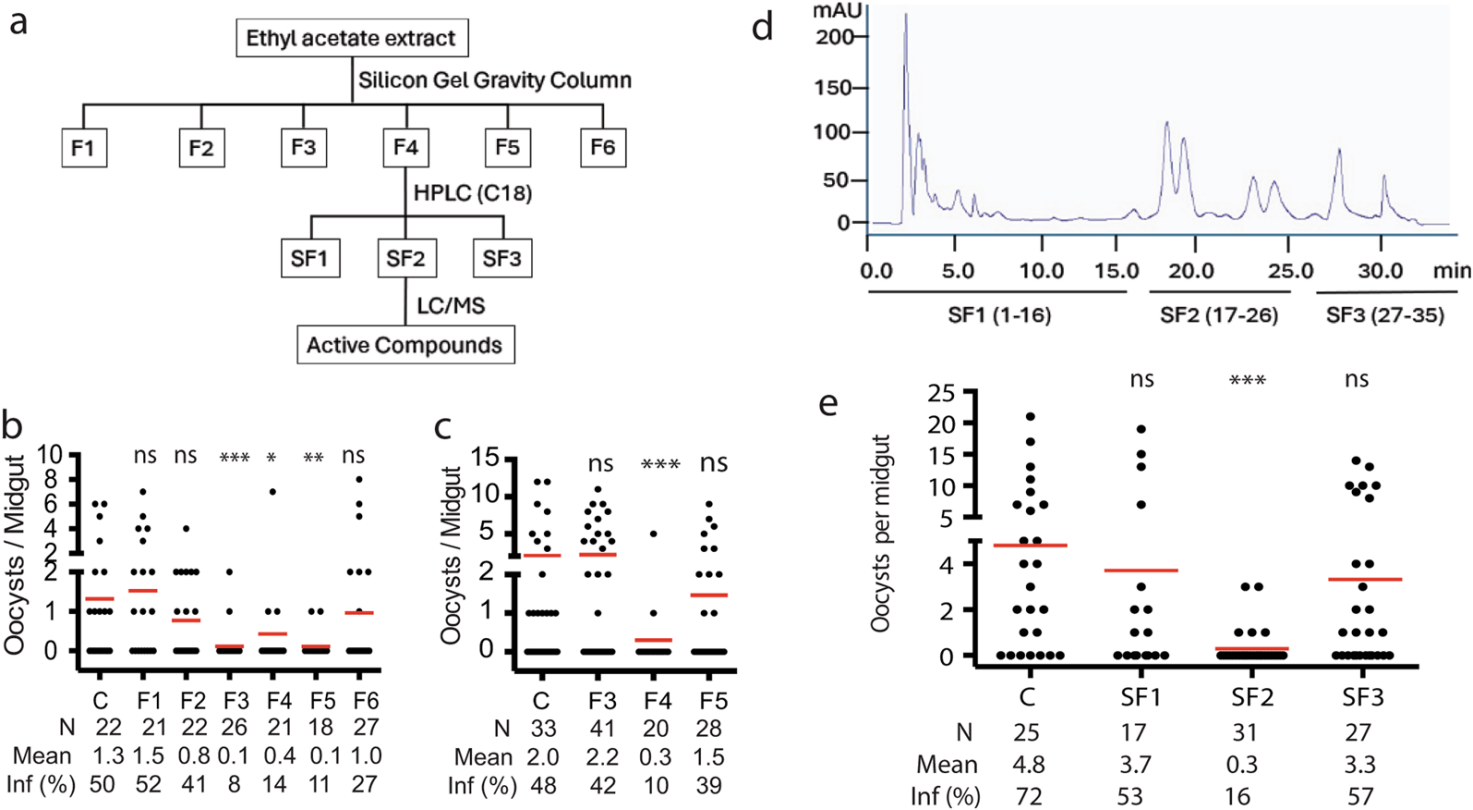
Isolation of active fractions through chromatography. **a** Outline of the isolation procedure. **b** Pre-exposure of fractions by the spray assays (10 mg/m^2^) to mosquitoes affected *P. falciparum* infection differently. **c** When the spray concentration was reduced to 5 mg/m^2^, only fraction F4 was able to reduce the oocyst density in the midgut significantly compared with the control (*P* = 0.0001). **d** Fraction F4 was purified with semi-preparative HPLC using a gradient solvent of acetonitrile and water (0.2% TFA) from 10% to 100% acetonitrile in 35 min. The eluted fractions based on the different retention times were collected and named SF-1 to SF-3, respectively. **e** The malaria-blocking activity of fractions (5 mg/m^2^). *ns* not significant; *P < 0.05, **P < 0.005, ***P < 0.0001. In b, c, and e *N*: # of mosquitoes; *mean* average number of oocysts/midgut, *inf(%)* percentage of infected mosquitoes

Next, we sought to identify active metabolites in the extract that possess TB activity through external contact. About 20 g of the *P. lilacinum* extract were fractioned sequentially in a silica gravity column with hexane, dichloromethane (DCM), DCM/methanol mixtures, and methanol, generating six fractions, F1, F2, F3, F4, F5, and F6, respectively. Each fraction was fourfold of the silica volume (Fig. 3a). The contact-wise TB activity of these fractions was determined by the spray exposure approach described above. Results showed that fractions F3, F4, and F5 significantly reduced the oocyst intensity in the midgut compared with the control at 10 mg/m^2^ (Fig. 3b). Further testing of these fractions at a reduced concentration of 5 mg/m^2^ revealed that only fraction F4 possessed a significant TB activity and reduced midgut oocyst intensity by 94% (Fig. 3c). This result prompted us to further fraction F4 with semi-preparative HPLC using a linear gradient solvent of acetonitrile/water (0.2% TFA) with 50–100% acetonitrile. Three eluted fractions (SF1, SF2, and SF3) were collected on the basis of the retention time, and their TB activities were evaluated using the spray-exposure method (Fig. 3d). The results showed that the SF-2 at 5 mg/m^2^ significantly reduced oocyst intensity (Fig. 3e).

### Identification of the active compounds

**Fig. 4:**
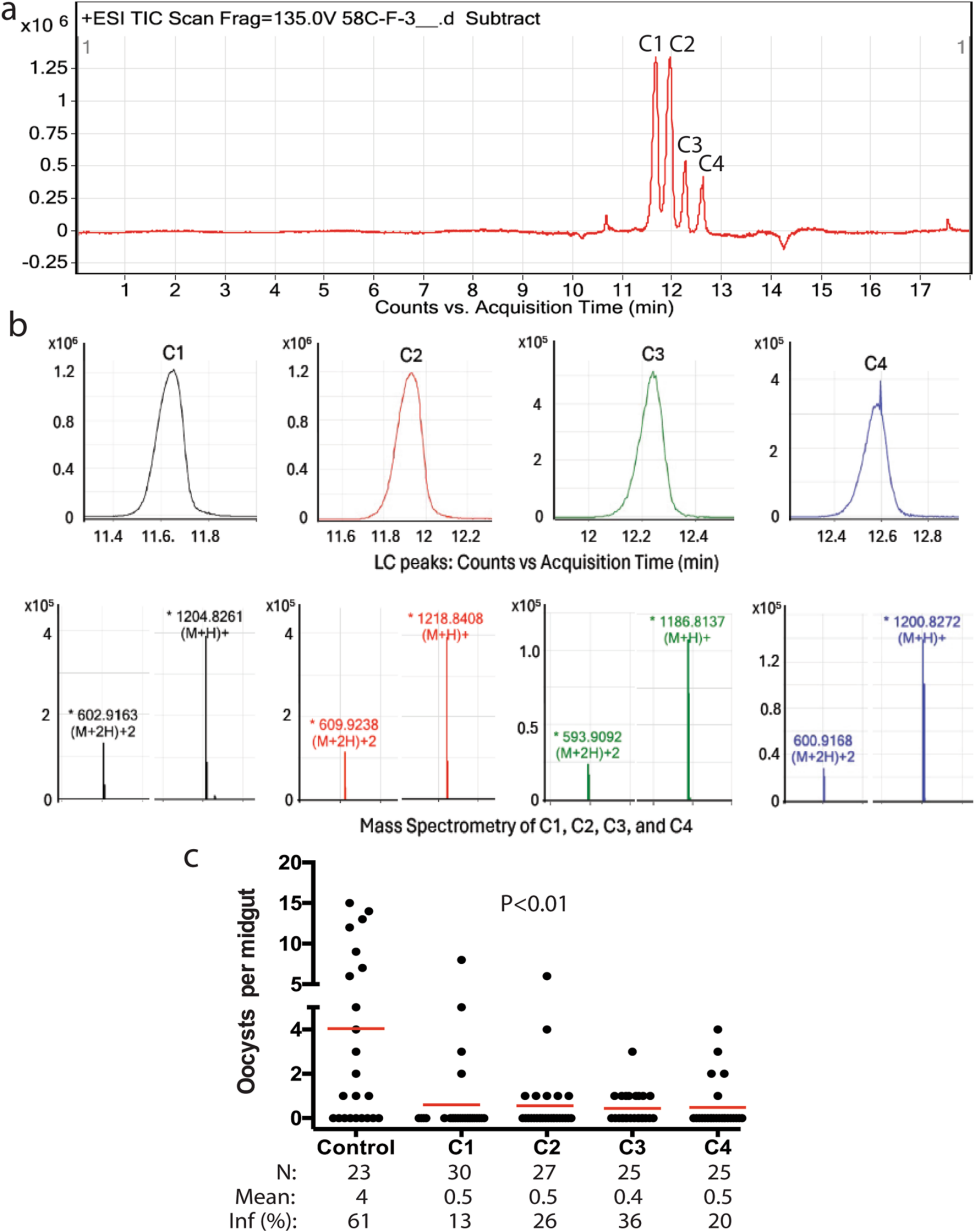
Identification of active compounds. **a** LC–MS profile of SF2 displaying four major peaks: C1, C2, C3, and C4. **b** Positive modes of the four peaks’ mass spectra shows M + H^+^ and M + 2H^+^. **c** Four compounds (C1, C2, C3, and C4) at 5 mg/m^2^ inhibited *P. falciparum* infection in mosquitoes by direct contact. *N* # of mosquitoes, *inf(%)* percentage of infected mosquitoes

LC–MS analysis of the SF-2 fraction revealed four peaks, C1, C2, C3, and C4 (Fig. 4a). The mass spectrum of the four peaks indicated that each contained one major compound with two major ionized forms, M + H^+^ and M + 2H^+^ (Fig. 4b). Considering the molecular mass of proton as 1.0073 daltons, the precise molecular masses of C1, C2, C3, and C4 were 1203.8189, 1217.8335, 1185.8064, and 1199.8199 daltons, respectively. The four peaks were collected, dried, dissolved in acetone, and tested for contact TB activity at 5 mg/m^2^. The results showed that all four compounds displayed significant contact TB activity (*P* < 0.01, Fig. 4c).

**Fig. 5:**
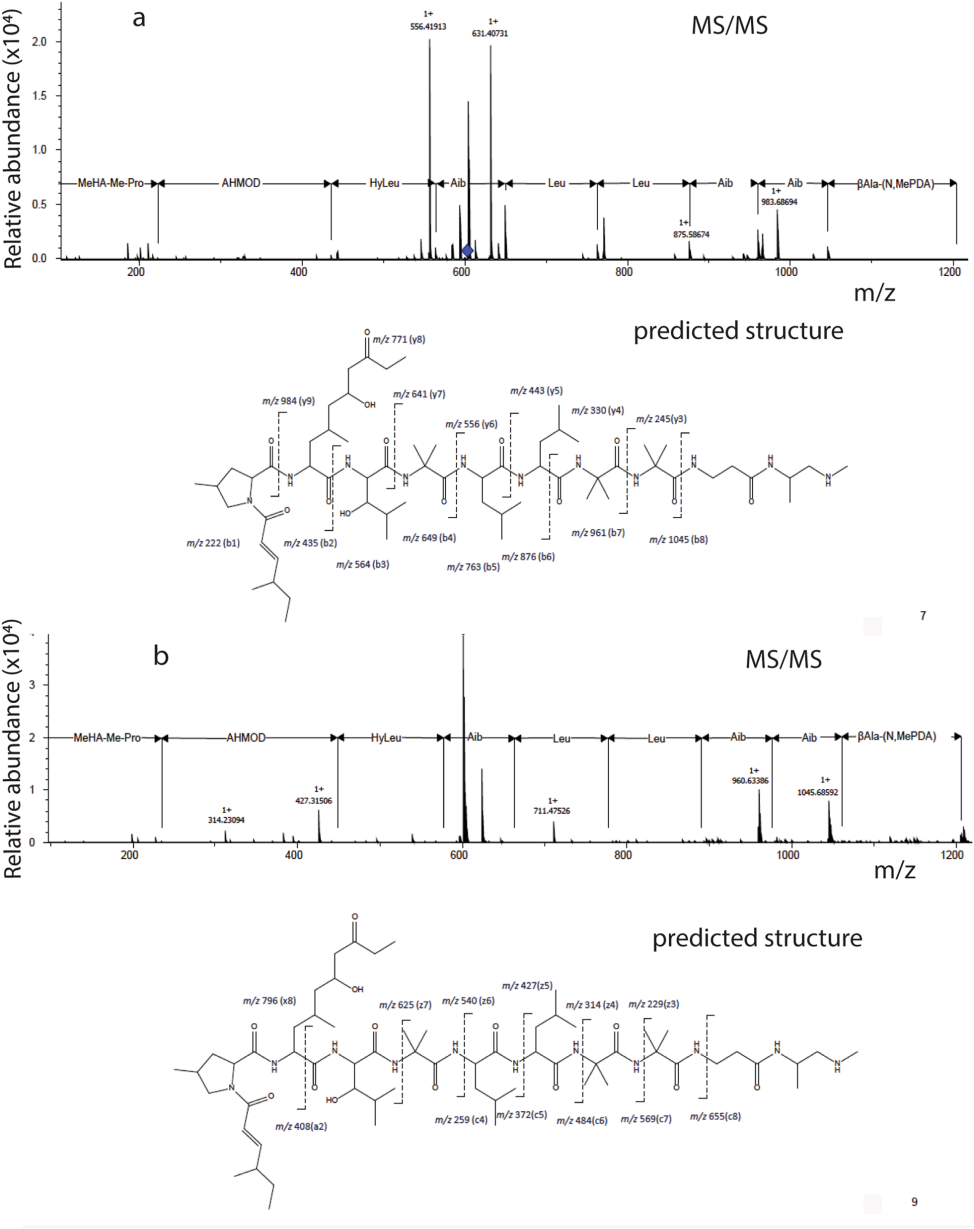
C1 was identified through tandem mass spectrometry as leucinostatin B by two approaches. **a** Collision-induced dissociation (CID) profile of C1 in the MS/MS analysis and the predicted corresponding structure. **b** Electron capture dissociation (ECD) profile of C1 in the MS/MS analysis and the predicted corresponding structure

**Fig. 6:**
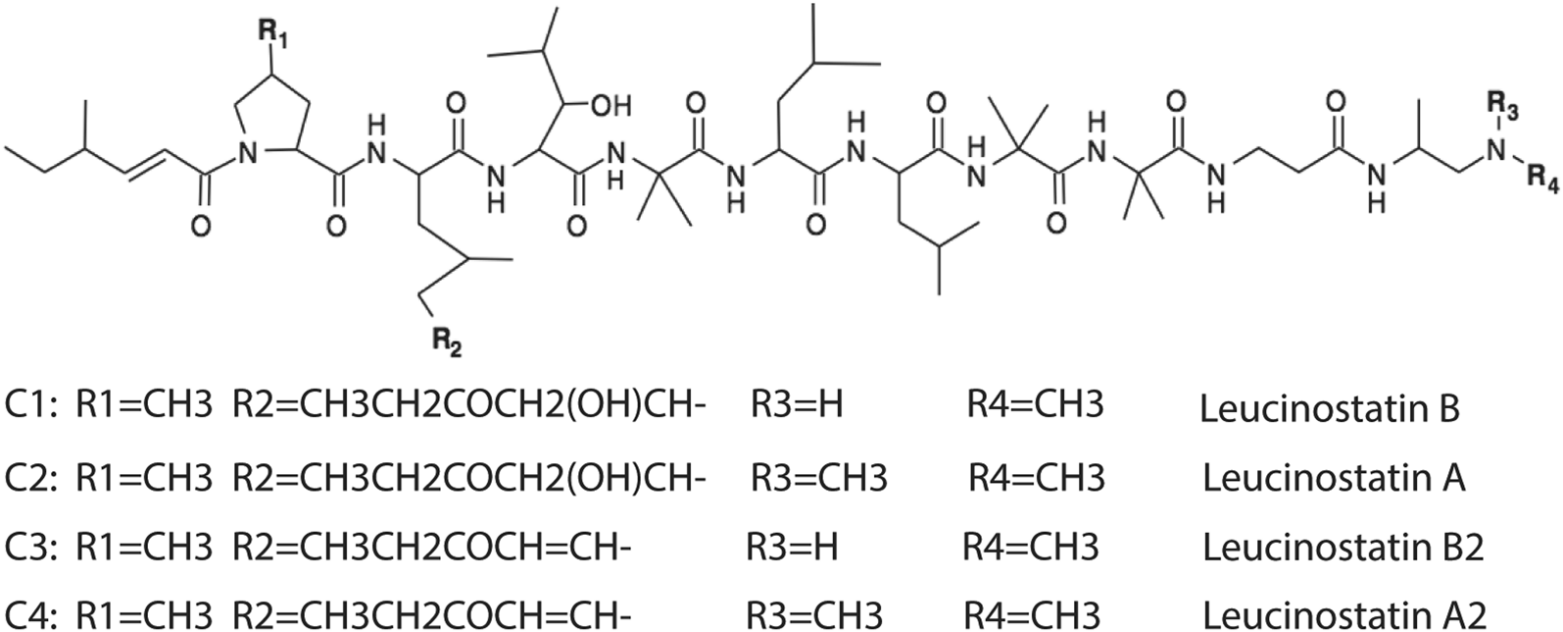
Structures of the four active compounds were determined as different derivatives of leucinostatins. The compound of C1, leucinostatin B, contains the substituent –CH3 in position R1, R2=CH3CH2COCH2(OH)CH-, R3 = –H and R4 = –CH3 while the compound of C3, leucinostatin B2, only has the different substituent –CH3CH2COCH=CH– in position R2. Another difference exists at position R3, which is observed with the substituents -CH3 in the compound of C2 and C4 (leucinostatins A and A2) compared with C1 and C3, respectively

To identify the chemical structures of these active compounds, they were subjected to analysis using tandem mass spectrometry (MS/MS). The MS/MS ion spectrum of C1 was determined to be leucinostatin B by collision-induced dissociation and electron capture dissociation (ECD) (Fig. 5). Given that C1–C4 have similar features, we searched the identified compounds in literature [24] on the basis of their molecular masses and fungal species and determined C2, C3, and C4 to be leucinostatin A, leucinostatin B2, and leucinostatin A2, respectively. The exact calculated molecular mass of leucinostatin A, B2, and A2 are 1217.8363, 1185.8055, and 1199.8257, respectively, matching our detected mass data well. These four active compounds are linear nonribosomal polypeptides (Fig. 6). The C1 compound, leucinostatin B, contains the substituent -CH_3_ in position R_1_, R_2_ = CH_3_CH_2_COCH_2_(OH)CH-, R_3_ = –H and R_4_ = –CH_3_, while the C3 compound, leucinostatin B2, only has the different substituent -CH_3_CH_2_COCH = CH- in position R_2_. Another difference exists at position R_3_, with the substituent –CH_3_ in C2 and C4 (leucinostatins A and A2) compared with C1 and C3, respectively.

### Contact-wise TB activity of leucinostatin A

**Fig. 7:**
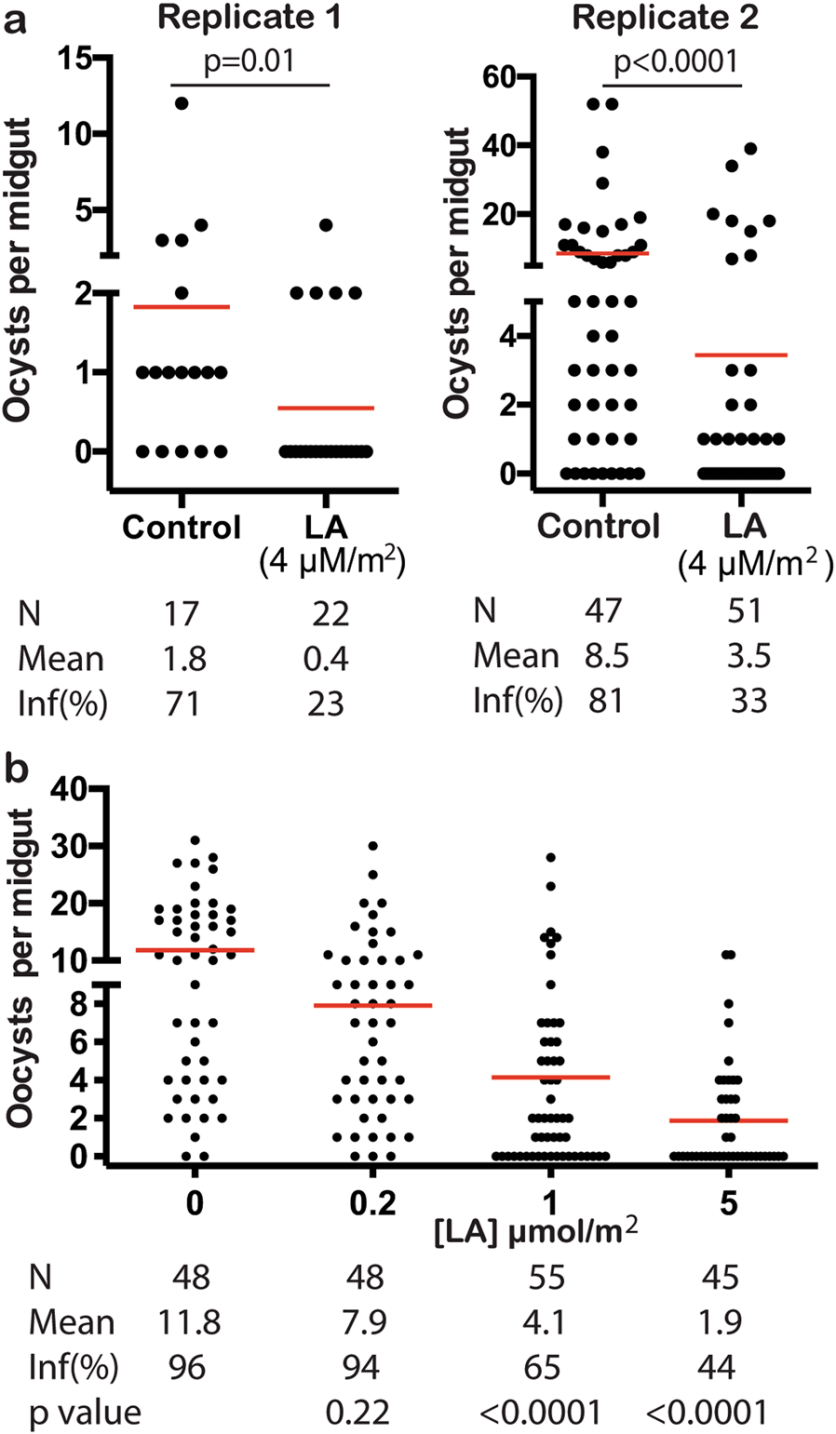
Leucinostatin A spray inhibited *P. falciparum* infection in *An. gambiae* in a dose-dependent manner. **a** Leucinostatin A spray was confirmed to reduce *P. falciparum* oocysts in *An. gambiae* midgut transmission at 5 µmol/m^2^ in two replicates. **b** Serial dilutions of leucinostatin A spray at 5, 1, and 0.2 µmol/m^2^ were examined for their TB activity. The EC_50_ dose was calculated as 0.59 µmol/m^2^. *N* # of mosquitoes, *mean* average number of oocysts/midgut,* inf(%)* percentage of infected mosquitoes

Next, we examined the contact-wise TB activity of leucinostatin A in detail. First, we repeated the pre-exposing mosquitoes with leucinostatin A spray (5 mg/m^2^ or 4 μmol/m^2^) on *P. falciparum* transmission to *An. gambiae* with different infection intensities. The results confirmed the leucinostatin A spray (4 μmol/m^2^) significantly limited *P. falciparum* transmission to mosquitoes in two treatments (Fig. 7a). The oocyst density was reduced to 0.4 and 3.5 oocysts/midgut in leucinostatin A-exposed mosquitoes as compared with that in the controls (1.8 and 8.5 oocysts/midgut, respectively) in two independent experiments. Moreover, leucinostatin A exposure also significantly reduced mosquito infection prevalence by 48% (Fig. 7a). Then, using serial dilutions of leucinostatin A, we showed dose-dependent TB activity of leucinostatin A spray, and EC_50_ was calculated to be 0.59 µmol/m^2^ or 0.7 mg/m^2^ (Fig. 7b).

**Fig. 8:**
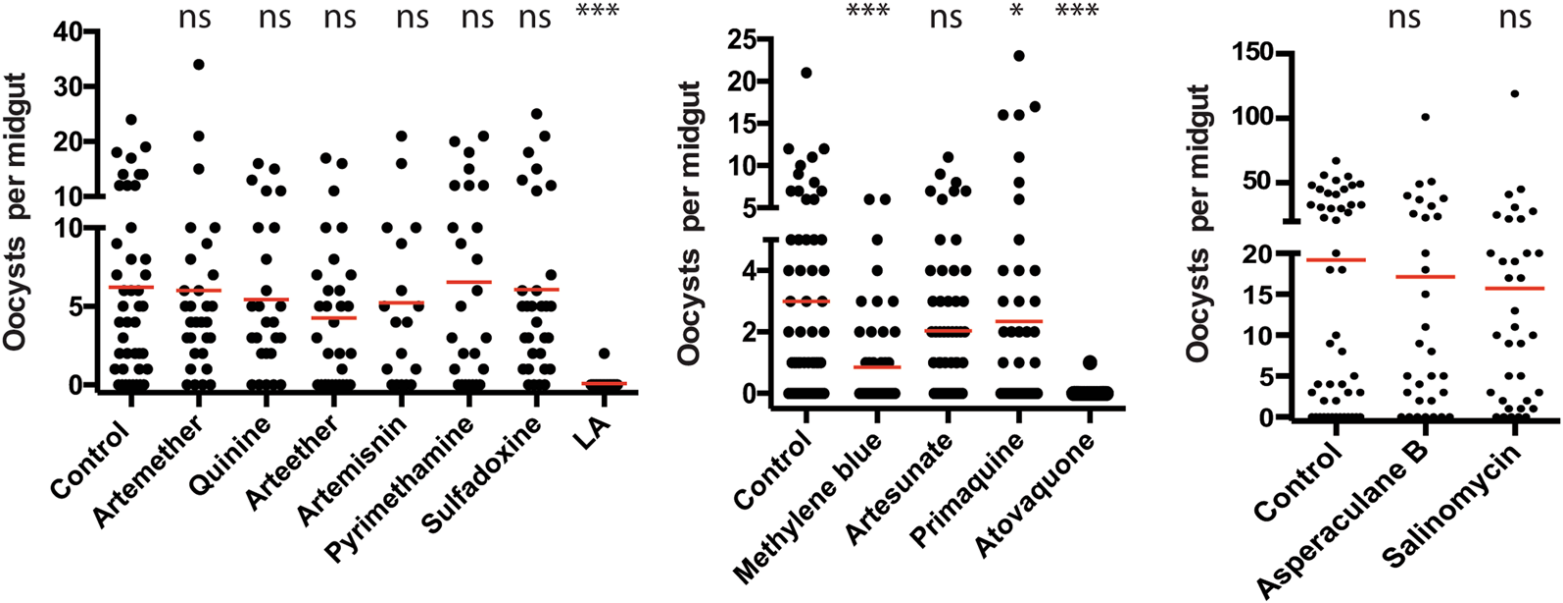
Antimalarial drugs and chemicals were examined for their contact-wise TB activity. A collection of oral antimalarial medicines at 0.1 mmol/m^2^ were pre-exposed to mosquitoes for 1 h and then the mosquitoes were fed with *P. falciparum* containing human blood by SFMA. The tested compounds include quinine, primaquine, artemisinin and its derivatives (artemether, arteether, artesunate), pyrimethamine, sulfadoxine, methylene blue, salinomycin, and asperaculane B. The oocyst densities of individual compounds were compared with those of the control. *ns* not significant; *P < 0.01, **P < 0.001, ***P < 0.0001

Using the spray-exposure approach, we compared the contact-wise TB activity of leucinostatin A with other antimalarial compounds that have different mechanisms of action against parasites (Supplementary materials). At the dose of 100 μmol/m^2^, methylene blue, atovaquone, and leucinostatin A significantly limited *P. falciparum* transmission to mosquitoes (*P* < 0.01, Fig. 8). Specifically, methylene blue at this concentration inhibited infection prevalence by 48.3% compared with the control, whereas leucinostatin A and atovaquone sprays completely blocked malaria transmission to mosquitoes. The results are summarized in Table 1.

**Table 1:** Contact-wise TB activity of antimalaria compound sprays (100 μM/m^2^)

Compounds	Reduction in oocyst number/midgut (%)	TB activity (%)	*P*-value to the negative control
Leucinostatin A	98.7	94.8	** < 0.0001**
Quinine	12.5	0	0.83
Primaquine	23.3	41.2	**0.015**
Artemisinin	15.8	9.2	0.47
Artemether	3.2	0	0.89
Arteether	31.3	18.9	0.16
Artesunate	33.3	19.8	0.32
Pyrimethamine	0	8.1	0.95
Sulfadoxine	2.2	0	0.92
Methylene blue	71.7	48.4	**0.0002**
Asperaculane B	10.9	2.7	0.73
Salinomycin	18.2	0	0.62
Atovaquone	99.3	96.8	< 0.0001

### Inhibition of *P. falciparum* at different developmental stages by leucinostatin A

**Fig. 9:**
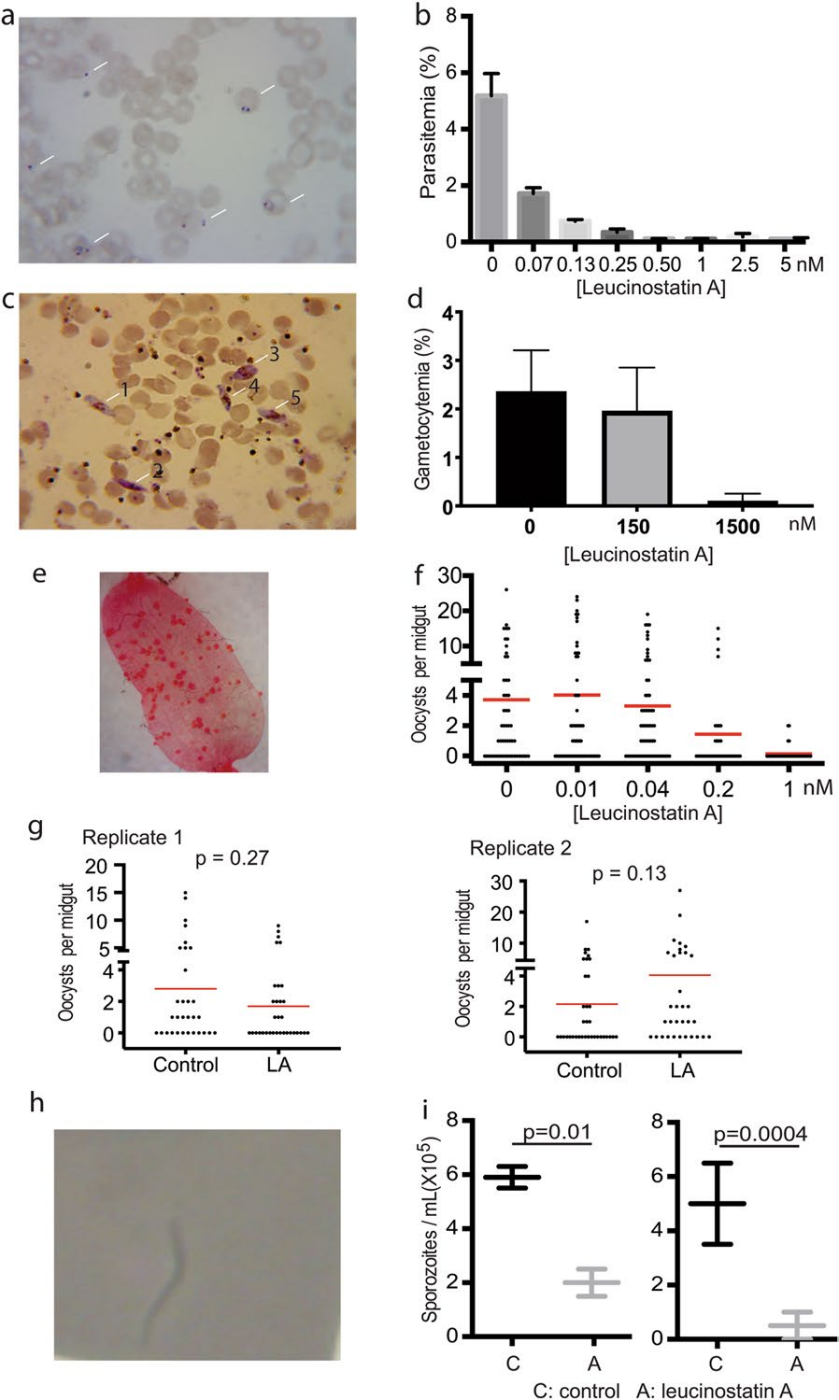
Leucinostatin A affected multiple stages in *P. falciparum* development. **a** The parasitemia of the infected red blood cells with *P. falciparum* was determined by counting the RBC infected with the parasite, and the parasites were stained with Giemsa staining as blue color. **b** Leucinostatin A inhibited asexual stage *P. falciparum* development in a dose-dependent manner with an EC_50_ of 0.05 nM. **c** The gametocytemia was determined by counting the Giemsa-stained gametocytes. Numbers mark the gametocytes. **d** Leucinostatin A inhibited the development of gametocytes with the EC_50_ of 220.5 nM. **e** Mosquito midguts infected with *P. falciparum* oocysts. Red dots are oocysts. **f** Leucinostatin A inhibited *P. falciparum* infection in mosquitoes by feeding with an EC_50_ of 0.16 nM. **g** Leucinostatin A did not interfere with the development of oocysts after oocysts had formed in midguts. **h** Live sporozoites examined under a light microscope. **i** Leucinostatin A significantly reduced the live sporozoites in mosquito midguts (*P* < 0.05)

Finally, we evaluated the activity of leucinostatin A against *P. falciparum* at different developmental stages. First, we analyzed leucinostatin A against asexual erythrocytic development of *P. falciparum*. Synchronized parasites (Fig. 9a) were exposed to leucinostatin A for 72 h with a changing medium containing the same concentration of drugs every 48 h. Results showed that leucinostatin A exhibited a dose-dependent inhibition of asexual *P. falciparum* development with an EC_50_ value of 0.05 nM (Fig. 9b).

To determine the effects of leucinostatin A on gametocytogenesis, *P. falciparum*-iRBC without any gametocytes was diluted with uninfected RBC infection with 0.5% parasitemia. On day 4, leucinostatin A was added, and the medium was changed daily with the fresh medium containing leucinostatin A. On day 14, the gametocytemia was determined by counting the Giemsa-stained gametocytes (Fig. 9c). At 1.5 μM, leucinostatin A almost completely limited the development of gametocytes (Fig. 9d).

Next, we examined leucinostatin A for its TB activity in blood by feeding. Leucinostatin A in DMSO was mixed with *P. falciparum*-infected blood at concentrations from 0 to 1 nM and fed to *An. gambiae* using SMFA to count oocysts (Fig. 9e). Results showed that leucinostatin A, when fed to mosquitoes, displayed potent transmission-reducing activity, with an EC_50_ of 0.16 nM (Fig. 9f).

We further evaluated the effect of leucinostatin A on sporogonic development. First, we infected *An. gambiae* with *P. falciparum* through membrane feeding. About 24 h after infection, when the ookinetes had developed into oocysts, we fed mosquitoes with 10% sugar containing 100 nM leucinostatin A daily until day 7. At this concentration, we did not observe significant differences in oocyst morphology and number between the leucinostatin A-treated group and the control (*P* > 0.5) (Fig. 9g), indicating that leucinostatin A did not inhibit oocyst development.

Finally, we examined the effects of leucinostatin A on sporozoite development. After the infection of *An. gambiae* with *P. falciparum*, mosquitoes were fed with a sugar solution containing either 1 µM of leucinostatin A or the DMSO vehicle on day 10 and day 11 post-infection, and sporozoites were enumerated on day 12 (Fig. 9h). The results showed that leucinostatin A significantly reduced the number of salivary gland sporozoites by 66% and 90% in the two replicates (*P* < 0.05) (Fig. 9i). These results collectively demonstrate that leucinostatin A is an anti-*P. falciparum* compound acting on multiple developmental stages in the human host and mosquitoes, most likely targeting parasite invasion-related components.

## Discussion

Natural products are ideal sources of bioactive molecules against malaria parasites. For example, two potent antimalarial drugs, quinine and artemisinin, both come from plants. Previously, we isolated *p*-orlandin from *Aspergillus nigri*[16], Asperaculane B from *A. aculeatus* [18], pulixin from *P. lilacinum* [20], and Sterigmatocystin from *Penicillium janthinellum* [17] that have malaria TB activity.

Pre-exposing *An. gambiae* to *P. lilacinum* acyl acetate extract blocked *P. falciparum* oocyst development in mosquito midguts [20]. Here, we isolated and identified leucinostatin A, B, A2, and B2 as the active compounds that blocked malaria transmission to mosquitoes via an external contact-wise approach. Although leucinostatins have been reported to inhibit erythrocytic stage malaria [25], they have not been reported against malaria transmission to mosquitoes, let alone via pre-exposure spray.

Transmission blocking through contact is a new approach to malaria control [20, 26]. Direct contact exposure of leucinostatin A to mosquitoes disrupts the parasite lifecycle at both the ookinete and sporozoite stages, which opens new avenues for developing TB agents through perturbing parasite biology in the mosquito rather than the human host. Leucinostatins could be administered in a way that mimics contact with an insecticide on a bed net [27]. Directly applying fungal extract into the environment to control malaria is cheap, fast, and effective in preventing malaria in endemic areas where poverty is widespread and the cost is the limitation factor. The EC_50_ of leucinostatin A in inhibiting malaria transmission by direct contact is 0.59 µmol/m^2^ or 0.7 mg/m^2^. The inhibition efficiency of leucinostatin A is similar to atovaquone and better than other examined compounds [26]. Leucinostatins also have antimicrobial activity against various bacteria species with EC_50_ of between 2.5 μM and 100 μM [28] and some fungal strains with minimal inhibitory concentrations between 10 μM and 25 μM [29].

Leucinostatins belong to a class of non-ribosomal peptides, peptaibiotics, and 24 different leucinostatins have been reported [24]. Leucinostatins contain a high proportion of unnatural amino acids. This unique composition of the peptides makes leucinostatins more hydrophobic than regular peptides and tends to interact with cell membranes [30]. The self-aggregation of leucinostatin A was observed in the lipid bilayer, leading to lipid phase transition and membrane fluidity [31], resulting in murine leukemic membrane lysis and complete cell growth inhibition at 0.5 µg/mL [32].

The mechanisms of leucinostatins against protozoan parasites have also been investigated [25]. Leucinostatin A forms an ionophore in liposomes, consistent with peptide-induced translocation from the inner to the outer leaflet of liposomes [31]. Notably, leucinostatin A induces loss of mitochondrial membrane potential [25]. The general cytotoxicities of leucinostatin A vary with different cell lines, including MRC-5 (human fetal lung fibroblast cells, 2550 ng/mL) [33], HeLa cells (50 ng/mL) [34], and DU145 in monoculture (prostate cancer cells, > 1 µg/mL). At the organism level, in vivo toxicity of leucinostatin A to mammals has been reported. LD_50_ of leucinostatin A to mice by the intraperitoneal route and oral were 1.6 mg/kg [34] and 5.4 mg/kg [35], respectively. Acute toxicity of leucinostatin A has also been observed in mice by the intraperitoneal route [31]. The external toxicity of leucinostatins to vertebrate animals has not been reported yet. It is worth noting that the general cytotoxicity to human cells is > 400-fold higher than that to *Plasmodium* parasites, highlighting the great potential of leucinostatin A as a novel antimalarial.

We show that leucinostatin A efficiently inhibited asexual *P. falciparum* and the gametocyte development and significantly reduced the number of oocysts in midguts and the number of sporozoites in salivary glands. However, leucinostatin A could not stop the growth of oocysts after the oocyst had formed. These data suggest that leucinostatins target parasite invasion-related components such as mitochondria, which is consistent with the mechanisms of action for leucinostatins [25] since the invasion is an energy-consuming process.

## Conclusions

We elucidated the contact-wise approach to control malaria transmission and found leucinostatins as effective malaria-blocking small molecules via contact. While most antimalarials do not have contact-wide TB activity, leucinostatin A has this activity, which was as potent as atovaquone and significantly stronger than other tested small molecules. In addition, the selectivity index of leucinostatin A to asexual-stage parasites and ookinetes over human cells exceeds 400 and 1000 folds, respectively, suggesting that *P. lilacinum* fungal metabolite spray possesses excellent potential to stop malaria transmission.

## Supplementary Information

Supplementary material 1.

## Data Availability

All data generated or analyzed during this study are in this published article.

## Abbreviations

LA: Leucinostatin A
SMFA: Standard membrane feeding assay
UHP: Ultra-high-performance
LC: Liquid chromatography
MS: Mass spectrometry
TB: Transmission-blocking
