# Supplementary material for: Leucinostatins from fungal extracts block malaria transmission to mosquitoes
Source: Parasit Vectors. 2024 Sep 20;17:401. doi: 10.1186/s13071-024-06450-y (PMC11414030; doi:10.1186/s13071-024-06450-y)
Supplement: Supplementary file 1 — Supplementary material 1. [file 13071_2024_6450_MOESM1_ESM.docx]

**Table 1. Inhibition activities of antimalaria compounds** **against each sexual stage parasite development**

| Compounds | MW | Inhibition activities against each sexual stage (EC_50_, µM) | | | | | | Ref |
| --- | --- | --- | --- | --- | --- | --- | --- | --- |
|  |  | EG | LG | MIC | MAC | Ooc^d^ | SPZ |  |
| quinine | 324.4 |  | 90% ^a^ |  |  | 1.9 |  | [1] |
| primaquine | **259.4** | 25-55% ^a^ | 25-55% ^a^ | 15% ^a^ | 18% ^b^ | 0.7 |  | [1]^,^ [2] |
| artemisinin | **282.3** | <0.0001 | <0.001 | 0.224 | 11% ^b^ |  |  | [2]^,^ [3] |
| artemether | **298.4** |  |  | 46% ^a^ | 10 % ^b^ |  |  | [2] |
| arteether | 312.4 |  | 10 mg/kg ^c^ |  |  |  |  | [4] |
| artesunate | 384.4 |  | 39% ^a^ | 0.483 | 10 % ^b^ | 0.0003 |  | [1]^,^ [2] |
| pyrimethamine | 248.7 |  |  | 0.0087 | 7 % ^b^ |  |  | [2] |
| sulfadoxine | 314.6 |  |  |  |  |  |  | [5] |
| methylene blue | 319.9 | 0.015 | 0.03 | 0.201 | 0.904 | 0.023 |  | [2] |
| salinomycin | 751.0 | 0.030 | 0.014 |  |  | 0.018 | 0.035 | [6] |
| asperaculane B | 237.1 |  |  |  |  | 0.0079 |  | [7] |
| atovaquone | 366.8 | 0.0075 | 0.0075 | 0.012 | 0.028 |  |  | [2] |

**Note**: ^a^: inhibition rate at >5 µM of the drug; ^b^: inhibition rate at 0.5-5 µM of the drug; ^c^: *In vivo* study. MW: molecular weight. ^d^: inhibition of a compound on the transmission of parasites to mosquitoes. EG: early-stage gametocyte; LG: late-stage gametocyte; MIC: micro gamete; MAC: macro gamete; SPZ: sporozoites; Ooc: oocyst intensity by SMFA; Ref: Reference.

**Reference**:

1. Chotivanich K, Sattabongkot J, Udomsangpetch R, Looareesuwan S, Day NP, Coleman RE, et al. Transmission-blocking activities of quinine, primaquine, and artesunate. Antimicrob Agents Chemother. 2006;50 6:1927-30; doi: 10.1128/AAC.01472-05. <http://www.ncbi.nlm.nih.gov/pubmed/16723547>.

2. Adjalley SH, Johnston GL, Li T, Eastman RT, Ekland EH, Eappen AG, et al. Quantitative assessment of Plasmodium falciparum sexual development reveals potent transmission-blocking activity by methylene blue. Proc Natl Acad Sci U S A. 2011;108 47:E1214-23; doi: 10.1073/pnas.1112037108. <http://www.ncbi.nlm.nih.gov/pubmed/22042867>.

3. Kumar N, Zheng H. Stage-specific gametocytocidal effect in vitro of the antimalaria drug qinghaosu on Plasmodium falciparum. Parasitology research. 1990;76 3:214-8. <http://www.ncbi.nlm.nih.gov/pubmed/2179946>.

4. Tripathi R, Dutta GP, Vishwakarma RA. Gametocytocidal activity of alpha/beta arteether by the oral route of administration. Am J Trop Med Hyg. 1996;54 6:652-4. <http://www.ncbi.nlm.nih.gov/pubmed/8686787>.

5. Villa M, Buysse M, Berthomieu A, Rivero A. The transmission-blocking effects of antimalarial drugs revisited: fitness costs and sporontocidal effects of artesunate and sulfadoxine-pyrimethamine. Int J Parasitol. 2021;51 4:279-89; doi: 10.1016/j.ijpara.2020.09.012. <https://www.ncbi.nlm.nih.gov/pubmed/33508331>.

6. D'Alessandro S, Corbett Y, Ilboudo DP, Misiano P, Dahiya N, Abay SM, et al. Salinomycin and other ionophores as a new class of antimalarial drugs with transmission-blocking activity. Antimicrob Agents Chemother. 2015;59 9:5135-44; doi: 10.1128/AAC.04332-14. <http://www.ncbi.nlm.nih.gov/pubmed/26055362>.

7. Niu GD, Hao Y, Wang XH, Gao JM, Li J. Fungal Metabolite Asperaculane B Inhibits Malaria Infection and Transmission. Molecules. 2020;25 13; doi: 10.3390/molecules25133018. <Go to ISI>://WOS:000550294000001.
